# Reorienting risk to resilience: street-involved youth perspectives on preventing the transition to injection drug use

**DOI:** 10.1186/s12889-015-2153-z

**Published:** 2015-08-19

**Authors:** Kira Tozer, Despina Tzemis, Ashraf Amlani, Larissa Coser, Darlene Taylor, Natasha Van Borek, Elizabeth Saewyc, Jane A. Buxton

**Affiliations:** British Columbia Centre for Disease Control, 655 West 12th Avenue, Vancouver, BC V5Z4R4 Canada; Faculty of Health Sciences, Simon Fraser University, 8888 University Drive, Burnaby, BC V5A1S6 Canada; School of Population and Public Health, University of British Columbia, 2206 East Mall, Vancouver, BC V6T1Z3 Canada; University of British Columbia School of Nursing, T201-2211 Westbrook Mall, Vancouver, BC V6T2B5 Canada

## Abstract

**Background:**

The Youth Injection Prevention (YIP) project aimed to identify factors associated with the prevention of transitioning to injection drug use (IDU) among street-involved youth (youth who had spent at least 3 consecutive nights without a fixed address or without their parents/caregivers in the previous six months) aged 16–24 years in Metro Vancouver, British Columbia.

**Methods:**

Ten focus groups were conducted by youth collaborators (peer-researchers) with street-involved youth (*n* = 47) from November 2009-April 2010. Audio recordings and focus group observational notes were transcribed verbatim and emergent themes identified by open coding and categorizing.

**Results:**

Through ongoing data analysis we identified that youth produced risk and deficiency rather than resiliency-based answers. This enabled the questioning guide to be reframed into a strengths-based guide in a timely manner. Factors youth identified that prevented them from IDU initiation were grouped into three domains loosely derived from the risk environment framework: Individual (fear and self-worth), Social Environment (stigma and group norms – including street-entrenched adults who actively discouraged youth from IDU, support/inclusion, family/friend drug use and responsibilities), and Physical/Economic Environment (safe/engaging spaces). Engaging youth collaborators in the research ensured relevance and validity of the study.

**Conclusion:**

Participants emphasized having personal goals and ties to social networks, supportive family and role models, and the need for safe and stable housing as key to resiliency. Gaining the perspectives of street-involved youth on factors that prevent IDU provides a complementary perspective to risk-based studies and encourages strength-based approaches for coaching and care of at-risk youth and upon which prevention programs should be built.

## Background

The United Nations defines street-involved youth as “any boy or girl… for whom the street in the widest sense of the word… has become his or her habitual abode and/or source of livelihood, and who is inadequately protected, supervised, or directed by responsible adults”[1]. In Canada, youth aged 16–24 years make up 20 % of Canada’s homeless population [2]. The homeless count in Metro Vancouver in 2011 identified 321 homeless youth under 25 years, 80 % of whom had been homeless for more than one month [3]. However, one Vancouver agency reported providing services to almost 1,500 street-involved youth aged 16–24 years each year [4], which may better reflect the hidden nature of this population.

Street-involved youth are more likely than youth in stable housing to use drugs and to initiate drug use earlier in life [5, 6]. They are also more likely to use drugs intravenously, which puts them at greater risk of adverse health outcomes, such as addiction and communicable infections including hepatitis C virus and HIV [7, 8]. Further, street youth are 11 times more likely to die of drug overdose and suicide than youth in the general population [9]. Recent estimates suggest that 20‐50 % of street‐involved youth inject drugs [10–12]. In a Vancouver-based study, injection drug use (IDU) was reported by 41 % of street-involved youth who used illicit drugs other than marijuana [13]. Studies from Montreal, Canada calculated an incidence rate of 6.8 - 8.2 per 100 person-years for street youth initiating IDU; in other words, 7 - 8 % of street youth start injecting drugs each year [14, 15]. Previous research suggests that street-involved youth make the transition from non-injection drug use to IDU for various reasons including exposure to IDU and social influence from street-involved peers [16] and sexual/intimate partners [17, 18] self-medicating for depression, childhood trauma, or other mental illnesses [19]; coping with homelessness [6]; and difficulties accessing treatment for drug use [20].

However, despite the plethora of risk factors mounted against street youth that make them vulnerable to transitioning to IDU, many do not. An important step in addressing and preventing IDU by street youth is recognizing that the transition to IDU is not inevitable for all “at‐risk” youth [21].

At-risk youth who avoid IDU, are often referred to as “resilient”. Resiliency is perhaps best understood as a person’s ability to navigate and negotiate psychological, social, cultural, and physical resources that sustain their well-being in the context of exposure to significant adversity [22]. Resilience research with marginalized adolescents in Canada identified seven key factors that youth must be able to access in order to experience resilience: the development of a desirable personal identity, experiences of power and control, experiences of social justice, access to supportive relationships, experiences of a sense of cohesion with others, adherence of cultural traditions and access to material resources [23].

A review of the literature found that the majority of resilience studies with street-involved youth focus on how youth survive on the streets without a specific focus on IDU prevention. Further, much of the research examining IDU among street‐involved youth focuses on risk factors for IDU initiation, and few studies examine the factors that may prevent youth from IDU initiation [7]. Therefore, the Youth Injection Prevention (YIP) project set out to investigate IDU prevention from a unique angle, namely resilience. The project framed its inquiry to identify protective (rather than risk) factors that youth perceive as preventing them from using IV drugs, and moreover foster resiliency. It is anticipated that findings from this study will help service providers working with street-involved youth to identify, encourage and provide access to factors that enable resiliency with an eye to preventing IDU initiation among this at-risk population.

## Methods

This was a qualitative descriptive study [24] to identify youth’s views of protective factors that prevent the transition to IDU. Qualitative description provides a comprehensive summary of events. “*Researchers conducting qualitative descriptive studies stay close to their data and to the surface of words and events*” [24]. The study was conducted between November 2009 and April 2010 in Metro Vancouver in British Columbia, Canada.

The YIP project employed six youth to work as research collaborators throughout the project. The youth collaborators were recruited from partner organizations and selected through an interview process; they were aged 18–24 years and most had personal experience of street involvement and/or illicit drug use [25]. The youth collaborators received qualitative research methods training from research team members including how to lead a focus group, sensitivity training and performed mock focus groups [25]. Inclusion of youth collaborators on the research team added a participatory spin to the project and resulted in benefits to both the project and the youth collaborators themselves. The youth collaborators helped develop the questioning guide, facilitated focus groups, took focus group observation notes, participated in open coding exercises and discussed key themes [25, 26].

### Participants & sampling

Youth were eligible to participate in the study, if aged between 16 and 24, were clients of organizations that provided services to street-involved youth and had spent at least three consecutive nights in the past six months without a fixed address or not with their parents or care giver. Although our participants included young adults (ages 19–24) the term youth has been selected to be consistent with other research.

Youth participants were recruited via purposive and snowball sampling techniques by partner organizations that provided services to street-involved youth. Each partner organization advertised the focus groups to clients using posters. Detailed recruitment invitations and consent forms were given to interested youth and to youth that the organizations thought would ensure diversity of age, ethnicity and gender of the participants at each site. If the youth were interested they signed up at the agency front desk for a prescreening interview and if eligible were invited to participate in the focus group. The partner organizations included those providing services specifically to Aboriginal and to lesbian, gay, transgender and queer youth, thus ensuring we heard the perspectives of the most vulnerable and marginalized youth. As this study was focused on factors that dissuade street youth from IDU, we invited youth who had never used drugs intravenously to share their perspectives. Written consent was obtained by the researchers prior to commencing the focus group.

Ethical approval was obtained from the Behavioral Research Ethics Board at the University of British Columbia; the ethics committee was aware that for youth under the age of 19 years consent was sought from a parent or care giver; however where this was not possible youth were considered emancipated and able to give informed consent.

### Data collection

Focus groups (FGs) were used to obtain a broad range of information and stimulate discussion and dialogue [24]. To create a safe environment with room for anonymity, FGs occurred at partner organizations which were familiar to the participants and youth were encouraged to share examples regarding their peers as well as their own lives. Each FG lasted approximately 90 min. Participants were provided with a $25.00 (CAD) honorarium, return bus transportation tickets and food.

A semi-structured questioning guide was developed by the research team including the youth collaborators. FGs were conducted by a youth collaborator, a second youth collaborator was assigned to be the note taker, and a senior researcher was also present. A debriefing occurred between the youth collaborators and senior researcher following each FG.

Despite the study’s intention to identify resiliency factors, a review of transcripts of the first few FGs noted that the questioning guide and scripted prompts were eliciting ‘risk and deficiency’ rather than ‘resilience and strength-based’ answers. Further, the focus group facilitators reported that some participants appeared frustrated and somewhat uncooperative, and eager to leave the FGs. Being mindful of participant safety as well as the aim of the study to identify protective factors and examples of resiliency, the research team including the youth collaborators revised the questioning guide.

The questioning guide changes included the addition of an opening question “What are some supportive and helpful things in your life”, and reframing of prompts such as: “Why do you think some youth decide to inject?” to “What are some positive things in a youth’s life that help them not to inject?” and “Why did you or others decide not to inject (when offered)?” to “What were the positive things in your life that influenced you or others to decline (when offered)?” These revisions were successful in both eliciting the kinds of protective factor information the research team was interested in, and keeping participants more positively engaged in the FGs. The original and revised FG guide can be found in the YIP project Final Report [27].

### Data analysis

All focus groups were audio recorded and transcribed verbatim. Identifying information was removed from the data to protect participant confidentiality. Thematic analyses of focus group data was conducted according to four steps [28]: immersion, coding, categorizing and generation of themes. Data analysis began during data collection; two researchers immersed themselves in the data by reviewing audio recordings and re-reading transcripts on an ongoing basis so that issues that were not clear could be re-examined in a subsequent focus group. Data were entered in QSR NVivo 8 to assist organization of the data. Through open coding, a new category is created for each meaning unit that doesn’t fit with a previously created category. Categories are kept as distinct and mutually exclusive as possible. Coding was conducted independently and then the two researchers met to discuss findings and reach consensus. When the focus groups were complete, i.e. saturation occurred and no new concepts emerged from the focus groups despite the diverse sample of participants, the research team held a ‘coding workshop’ with the youth collaborators through a paper-based exercise. This coding workshop provided the youth collaborators a new research skill and experience, informed the revision of the categorical scheme developed by the two researchers and contributed to the validation of the data [26].

Following two knowledge translation events with service providers and youth collaborators where preliminary data were shared and discussed, the research team revisited the categories identified as protecting against the transition to IDU. The categories were then grouped into conceptually related domains based loosely on the risk environment framework, which examines the interplay of various types of environments (social, physical, economic and political) and levels of risk (micro, meso, macro) in the prevention of HIV and reduction of drug-related harms [29–32]. In the context of harm reduction, the risk environment framework is helpful as it redistributes the focus of interventions and responsibility for drug harms from individuals to “something shared between individuals and social-economic structures” [32]. However as Sandelowski articulates, qualitative content analysis based on preexisting coding systems are always modified in the course of analysis to ensure the best fit of the data [33].

## Results

A total of 47 street involved youth participated in ten FGs. Participants were between the ages of 16 and 24 years with an average age of 21 years; 87 % were aged 19 years and older. Of the participants, 27 (57.4 %) were male, 19 (40.4 %) were female and 1 (2.1 %) identified as transgendered. Of those who reported their sexual orientation, 70.5 % reported being heterosexual, 9.1 % as gay or lesbian and 20.5 % as bisexual. Forty-five percent self-identified as Aboriginal, 34 % as Caucasian, 9 % multiethnic and 10 % as other ethnic groups. The drugs study participants reported using in the past month are shown in Table 1 and indicate poly-substance use; 62 % were using illegal substances other than marijuana.

**Table 1:** Drugs participants reported used in the past month

	Number	Percent
Tobacco	40	85.1
Alcohol	37	78.7
Marijuana	34	72.3
Ecstasy	17	36.2
Cocaine	16	34
Crack	13	27.7
Acid	13	27.7
Mushrooms	12	25.5
Heroin	10	21.3
Crystal methamphetamine	9	19.1
Speed	7	14.9
Other drugs	5	10.6

Seven categories of factors that protect against the transition to IDU were identified through the coding and grouped into three domains loosely derived from the risk environment framework: fear and self-worth (Individual), stigma, support/inclusion, family/friend drug use and responsibilities (Social Environment), and safe/engaging spaces (Physical/Economic Environment). Table 2 denotes the final categories and codes within each domain.

**Table 2:** Factors reported by street-involved youth to protect against the transition to injection drug use

Domain	Category	Codes
Individual	Fear	Fear of Needles
		Fear of Addiction
		Fear/Awareness of IDU Health Consequences: e.g. HIV, hepatitis C, overdose, physical appearance
	Self-Worth	Desire for Better Life/Goals
		Self Esteem
Social Environment	Stigma &Group Norms	Society’s negative views of IDU
		Peer Group negative view of IDU
		Adults who inject drugs deterring youth from IDU
	Support & Inclusion	Support from/Involved Families
		Membership to Peer Group/Community
		Positive Role Models
		Fear of Losing Peer Group/Family
		Connection with Culture/Communities
	Family/Friend Drug Use	Family Drug Use
		Observing Others
	Responsibilities	Responsibility for another Person e.g. child, or for a Pet
Physical Environment	Safe & Engaging Spaces	Opportunities for Recreation & Employment
		Housing (affordable/accessible)

*IDU* Injection drug use

### Individual level factors

Many of the reasons offered as to why youth choose not to inject drugs fell within an individual’s own locus of control, feelings, personal observations and preferences.

#### Fear

The notion of fear, aversion to needles and concerns regarding consequences of injecting were commonly noted as a major deterrent to IDU:

*“I’ve used the majority of the drugs out there. I’ve never injected… I know I’m scared shitless of needles and I don’t think I ever will” – FG7, #3*

*“I don’t like injection drugs because of the way, like, you have to, like, suck the blood out then and push it back in. That really grosses me out.” – FG1#1*

In addition to a fear of needles and injection practice itself, the youth mentioned a further fear of addiction which they saw as a down-hill trajectory, which almost inevitably followed the transition into IDU:

*“I just wouldn’t do that [IDU] cause that’s how people get really bad…I don’t want to end up on East Hastings… I want better for myself than that.” – FG4#1*

A few youth demonstrated considerable insight by citing their ‘addictive personalities’ as a reason to avoid injection drug use:

*“I personally wouldn’t do injection drugs ‘cause I have an extremely addictive personality so I’d mostly likely like it and continue to do it so that’s why I won’t do it.” – FG4#1*

Further to the broad fear of addiction, many youth demonstrated an understanding of the adverse health outcomes associated with IDU including transmission of viral infections, overdose and the physical appearance of those who inject. These concerns contributed to their decision to not use drugs intravenously:

*“I don’t inject because I don’t want to catch anything… If I was to fix I would be worried about catching HIV, Hep C, all that kind of stuff. ” – FG1#4*

*“A friend of mine asked me if I want to inject… I was, like, wait a second. I’ve never done this before and I don’t want to because I don’t know what the effects are, the outcome of this drug is… Or overdosing on the first time, you never know, you don’t know that you’re going to overdose on the first time” – FG5#2*

*“They have, like, holes in their face and they’re always scratching at themselves and there’s chunks of skin missing on their backs and on their necks and they just look dead.” – FG3#5*

#### Self-worth

It was evident that some of the youth in this study had a strong sense of self-worth and a desire for a better future which they perceived could be jeopardized by engaging in IDU. This is consistent with previous studies which suggest that youth with high levels of self-confidence, positive self-image and desire for a positive personal identity are less likely to engage in high-risk behaviours [34]. Many of the YIP participants credited their ability to avoid IDU with personal strength and satisfaction with their current lives:

*“I got scared and I realized, you know, I’m worth so much more than that. Like, I’m here on this earth for a reason and it’s not, you know, to be down there doing the Hastings shuffle…” – FG8#6*

*“I have lots of goals so I just work towards the goals. Education and a career” – FG6#1*

*“I just like my life how it is and I don’t want to throw it away for drugs.” – FG6#2*

One focus group participant's reflection on where his same-age mainstream peers were in their lives compared to him was motivation to avoid the transition to IDU:

*“I was thinking about all my friends that are successful and don’t do drugs and have jobs and just-- they’ve already made it in life and I’m just down there. I wouldn’t want to go any further down that bumpy road.” – FG7#1*

### Social environment factors

In addition to individual level protective factors, participants discussed in depth the ways that social environments can influence youths’ decisions to navigate away from IDU. Social environmental factors reported included negative perceptions such as stigma from society and their peer group, and observing the hardships of others who injected especially family and friends. However, the majority of the participants identified many positive factors such as supportive relationships, cultural connection and responsibility, which prevented the transition to IDU.

#### Stigma & group norms

Society’s negative views towards individuals who use injection drugs is generally problematic as it contributes to their marginalization and isolation; however, youths’ awareness of this stigma appeared to serve as a protective factor against the transition to IDU for youth who did not inject drugs.

*“A lot of people look down on, like, society big time looks down on injection drug use…I have a lot of friends who aren’t from this lifestyle at all and when they see one of my friends that use IV it’s almost like looking at a gremlin or something, you know. ” – FG5#1*

Peer group norms play a crucial role in shaping individual attitudes and behaviors [16]. Studies of street-involved youth in Vancouver and Montreal found that youth were more likely to inject drugs when their peers injected drugs, particularly because their peers who were newer to injection had yet to experience many adverse outcomes [35, 16]. Within the YIP study, in addition to noting societal stigma, the participants commented that IDU was looked down upon by their peer groups or that they simply preferred other forms of drug use:

*“Down here when people offer to doctor you or to inject you, it’s a real sign of disrespect.” –FG1#1*

In addition to society and peer groups, a few participants noted that social pressure to avoid IDU came from yet another group, adults who inject drugs. Examples were shared of street-entrenched adults actively discouraging youth from IDU and were viewed as protecting youth and concerned about their welfare:

*“Most people down there, when they see someone under 18, they’ll usually kick their ass or something and tell them to get lost, yeah… I see it daily all the time… kids coming down, they’re trying to get high and yeah, it doesn’t fly, man. They usually end up getting slapped around or fucking’ you know, they’ll grab a cop and say, listen, this kid’s fucking under 18, get him out of here, right?” – FG5#1*

This “code” of experienced adults who use drugs in Vancouver’s downtown eastside discouraging youth from initiating IDU has previously been reported [16]; although as noted by Small et al., it is likely a “convention [that] is routinely ignored”[16].

#### Support & inclusion

Participants commonly noted supportive and involved families as a protective factor against injection drug use:

*“I had a bad drug problem and she [my sister] made me go to detox as soon as I got out of the hospital. And then as soon as I got out of the detox she got me in to get [treatment] back home. And I went home to clean up for awhile, got a job right away and came back down here and been clean ever since.” – FG7#1*

*“I think the biggest part of it is when family and friends step in. ‘Cause then that’s when you realize that your friends and family actually do care about you. You’re not just a big shit that no one cares about.” – FG7#3*

Previous studies have shown that supportive relationships, in or outside the family, foster resiliency among at-risk youth; feeling loved, trusted, and having a sense of belonging can cultivate resiliency [36]. For many youth, the protective ‘sense of belonging’ came from peer groups with shared experiences and values:

*“Having, like, a sense of the family again and even if it’s just a fake family, you know, like, it’s not your real blood family, that helps a lot.” - FG10#1*

*“Like I said, mud- mud is thicker than blood. The street family’s definitely the greatest support. Some of them might be, you know, not the best but they’ll help you out whatever way they can.” – FG8#3*

The street family has previously been found to play a supportive role [37], although this may not be seen as fostering positive relationships by mainstream society. In addition to the support found in group affiliation, participants talked about the value of positive role models and mentors in preventing transition into IDU:

*“I have a mentor that I have a really good relation with who’s also, like, one of my best friends and she’s been there-- it’s really awesome. She’s helped introduce me to services around Vancouver to help me through whatever I’m going through. Also introduced me to my martial arts school and to various artists.” – FG7#1*

The support and sense of belonging to families, peer groups and communities was so valued that fear or experience of losing those relationships was also frequently mentioned as a reason not to initiate IDU:

*“Social factors, losing your friends and family…you might get to the point where you’re so addicted that the drug becomes your #1 priority and then you’re neglecting your family and your friends and then finally just drift apart…That’s a concern, yeah. Not a very positive thing.” – FG6#1*

Some youth also reported assessing the drug use behaviors of peer groups and actively seeking the company of people who did not inject.

*“Find something to do, go hang out with somebody who’s not in the wrong crowd. And find a good crowd to hang with.” FG9#2*

A number of Aboriginal youth also credited connections with their culture and communities as a protective factor:

*“Being sort of part of my culture, I-- not only do I dance, I also sing and I play a big role in-- throughout the community… We’ve developed a sense of other. And if you’re needing help, you could just go and talk to them.” - FG8#3*

*“I kind of grew up in foster care so I wasn’t, like, introduced to that stuff at all. And I thought it was really awesome and kind of like coming home, like, I was finally home and I knew who I was once I got in touch with that…I don’t think I’ll ever let it go.” - FG8#6*

#### Family/friend drug use

Some of the participants shared that IDU was common in their families and that they had been around the lifestyle for their whole lives. Further, many youth cited family deaths due to overdose or IDU related illness as reasons not to start using injection drugs.

*"If you’re going to grow up with it you want to be better than what you’ve grown up with or you’ve experienced.” – FG8#2*

*“Personally for me, like, my mom she died two years ago. She was an intravenous drug user for, like, 30 years -- pretty young but, like, when I see people her age and they look so young, like, healthy and young, like, my mom just looked, like, so haggard and old.” – FG1#3”*

In contrast to previous literature that largely reports family drug use as a risk factor for youth initiation [38, 39], the youth in our study were motivated to avoid IDU because of witnessing the challenges and consequences faced by family members with addiction. This illustrates well that resilience may not be determined solely by the presence or absence of certain risk factors, but rather determined by an individual’s ability to navigate life’s circumstances in a way that leads to optimal outcomes.

Other participants shared ‘cautionary tales’ based on their observations of friends, acquaintances or people on the street changing as a result of using drugs intravenously:

*“‘I’ve had about five friends lost to the Downtown Eastside. Like, they’re not dead or gone but their spirit is, like, they’re- they’re not the person that I used to know.” – FG2#6*

*“Just seeing the effects it has on other people. Like, Hastings and other areas, even going by on the bus, I mean, it’s only for a few seconds and you just, like, sort of glance over, it’s, like, hmm, it’s just for half a second you’re bound to see somebody that’s, like, doing the Hastings shuffle down the street. And it doesn’t look too appealing.” – FG8#4*

#### Responsibilities

The duty to protect and a moral responsibility to look after another life - be it child or relative was a factor cited by many youth participants that discouraged the initiation of IDU:

*“His kids are more important than him. He’s, like, either I get high or I feed my kids, what’s better? ” – FG7#4*

*“They’ll be drinking, drinking, drinking, party, party, party, oh, shit, I’m having a kid. Okay, now I gotta clean up, you know, and sometimes it lasts, sometimes it doesn’t and sometimes it bounces back“ – FG7#5*

*“Her mom’s sick and she’s going to die, like, next two or three years of something like that. And her mom just got back in contact with her and she just decided, like, for her mom’s sake that she doesn’t need to see her all sick and, you know.” – FG9#3*

### Physical & economic environment factors

#### Safe & engaging space

Safe spaces to hangout, skill building and job placement programs, and youth drop-in centres were commonly referenced as positive persuasions away from street life and injection drug use:

*“You need incentive to stay clean, like, stuff to do that they’re interested and, like, art programs, like, just anything that they could be there doing and not out getting high.” – FG1#3*

*“The only way that- that, like, Vancouver or even Canada, has a chance for young people, is places like < name of drop-in centre > where you can offer life skills. You can offer uhm..job placements, like, <name of program > because you get your money from not sucking dick or prostituting or whatever.” – FG3#2*

Before street-involved youth can benefit from the responsibility and meaningful engagement that employment and recreation opportunities provide, they must first encounter such opportunities. It is interesting to note that 25 % of the youth surveyed in the Metro Vancouver Homeless count indicated that they had been affected by the withdraw of youth services by one or more government agency [3]. Unsurprisingly, many YIP participants commented on the need for more or expanded youth programming:

*“There’s really not much to do in this city and it-- until you’re already hooked on drugs then people will come up and help you out, right. You know, so we do need, like, youth programming.” – FG8#5*

Youth, both with and without adequate, accessible and affordable housing commented on the crucial role that shelter plays in ones decision to use or avoid drugs. Many participants provided examples of using drugs as a means to keep warm during inclement weather, or to keep awake through the night to protect themselves and their belongings from harm on the streets.

*“I think in general most people do drugs because they’re on the streets and it makes it easier to stay on the freezing cold sidewalks at night, especially heroin.” – FG3#2*

Others commented that having a safe space to live was exceedingly helpful to avoid temptation or pressure to participate:

*“You know, if you’re in a shitty living situation, say on the street, where your stress level’s raised a lot more than what it would be if you had a nice place to live, you’re going to be more susceptible to doing things you wouldn’t normally do… making shitty decisions like picking up a needle or whatever.” – FG5#1*

The youth participants’ frequent mention of the value in safe, engaging spaces and opportunities for recreational and employment activities underscores the role that communities can play in providing avenues for resiliency for at-risk youth. Increasingly, youth programming and life skills programs have been shown to prevent youth drug use [34, 40]. Recreational and employment opportunities may also provide youth with skills that prevent IDU and foster resiliency such as how to cope with stress, solve problems effectively, build social support and connect with others [40, 41].

## Discussion

### Youth perspectives

As others have argued [42], it is important for service providers and policy makers to understand street youth’s perspectives on drug use in order to address the challenges in their lives. This study offered street-youth an opportunity to voice their concerns and assurances about their own health and wellbeing, as well as that of their peers. Study participants were keen to share their stories and experiences with the research team and provided many insights into how to prevent the transition of street‐involved youth to IDU and/or reduce drug‐related harms. Further, the involvement of youth collaborators as group facilitators enhanced the opportunity for rich data collection as our study population shared experiences with peers.

### Risk to resiliency

The intentional shift away from a risk framework to one of resilience in this study necessitated an early revision FG questioning guides to ensure that prompts and questions were eliciting information on resiliency and protective factors, rather than negative risk-based dialogues. Interestingly, youth in the first focus groups that elicited risk-based information were somewhat uncooperative and eager to leave the room, where as those in the later focus groups were more willing to stay and participate, some commenting that the experience of participating had been in many ways, therapeutic.

Through regular post FG debriefing and performing ongoing data analysis we were able to identify that participants were eliciting risk rather than resiliency based answers which enabled us to identify the need to revise the questioning guide in a timely manner. Despite the success in revising the questioning guides to elicit dialogue around factors that prevent IDU rather than cause it, the research team noted that many of the participants’ answers were still framed as negatives. For example, many participants discussed negative life events or experiences of friends and family as reasons to avoid IDU. We postulate that this is in part a result of these youth, like society at large, being accustomed to identifying risks, problems and deficits in their lives, rather than strengths and protective factors.

The observation that youth appeared primed to give risk-based and negative answers when asked about their drug use patterns and decisions, might prompt service providers to re-examine their own word choices and phrasing when asking youth questions. People who work with at-risk youth should be mindful to not only highlight risks and problems when conversing with the youth but where possible, ask questions that encourage strength-identification and positive possibilities.

### Rhodes’ risk environment

As mentioned, the “risk environment” framework has been increasingly used as a way to organize risk information in relation to the prevention of HIV and reduction of drug-related harms [29–32]. Discussing factors in terms of social and physical influences introduces a helpful shift of blame/credit, responsibility and opportunity from individuals to families, communities and society. Yet to date, few studies have taken an environmental approach to studying resilience in relation to drug use behavior. Therefore, acknowledging that risk and protective factors are often the inverse of each other, the risk environment framework was selected as a helpful starting point for categorizing the YIP data and contextualizing the reasons that youth provided that enable them to navigate away from IDU.

To better fit the YIP data, our categorical scheme took a slight departure from Rhode’s four ideal types of environments: physical, social, economic, and policy [31], as we added an “Individual” tier to account for the many internalized and personal factors that youth discussed, we merged the Economic and Physical environments and interestingly, did not find any content within our data that aligned with what Rhodes would consider the Political Environment. This is perhaps not surprising as our data was derived solely from the perspectives of young people who may not be aware of the policies and legal regulations that influence their experiences.

### Factors that promote resiliency and prevent IDU

The factors identified in our study that protect against the transition to IDU were categorized as: fear, self-worth, stigma, support and inclusion, family/friend drug use, responsibilities, and safe and engaging spaces. Although our findings are largely consistent with the dominant discourse in the field of IDU prevention, the spin of resilience elicited some new perspectives of the trajectory toward (and away from) IDU, not always seen in the literature.

#### Individual

Our participants demonstrated a considerable knowledge of the adverse health risks associated with injection drug use and frequently cited fear of needles, addiction, overdose, and compromised health as reasons governing their decisions to avoid injection drug use. This may indicate the success of education programs that go beyond “just say no” messaging. Participants also noted their desires for a better life and future goals as reasons not to experiment with injection drugs. The ability of the youth participants to attribute their present actions to their future well-being is worthy of pause and underscores the value of youth workers employing motivational interviewing techniques [43] to reinforce self-efficacy and discuss drug use behavior in the context of longer term goals and values.

#### Social

The youth in our study appeared to be influenced by a variety of social forces including societal stigma, peer group norms and interestingly, street-involved adults. While it is commonly noted that youth are influenced by the attitudes and behaviours of their peer groups, the comments regarding street-based adults who discourage youth IDU are somewhat novel. These street-involved adults could play an important role in IDU prevention and their cooperation/role in harm reduction interventions warrants further investigation. Previous work has shown that peer outreach workers are able to meet the youth ‘where they are’ and peers can serve as successful service providers [44]. The ‘Break the Cycle’ and “Change the Cycle” programs utilize a similar model where peers help prevent others from IDU initiation [45, 46].

Inclusion and the sense of belonging to families and peer groups was highly valued as a protective factor by participants, as was the support of positive role models and connections to cultural communities and practices. As previously noted in the literature [37, 47, 48], street youth’s ties to family and other social networks are key sources of resiliency upon which prevention programs should be built. In addition to receiving social support, the moral obligation to *provide* support to dependents was also discussed as a motivator to avoid IDU. Additionally, many youth referenced the negative experiences of friends and family members with IDU as reasons to avoid it themselves.

#### Physical

Lastly, participants emphasized the need for safe, affordable, stable housing to assist in the avoidance of IDU. Unger describes resiliency as a person’s ability to navigate and negotiate for resources to promote health [22]; however, resources must be accessible and available in order for youth to obtain them. Meeting youths’ basic needs, including housing security, can aid in the prevention of IDU as when resources run out, short term solutions like drug use can become the best or only coping mechanism [23]. Indeed, youth in our study described drug use when living on the streets as a means to provide shelter from the cold, reduce anxiety and create a sense of security in a very unsecure environment. Previous research has suggested that cumulative length of time youth spend without a consistent place to live is associated with an increased risk of an alcohol and/or illicit drug abuse disorder [49]. Further, shelter use has been strongly associated with use of other health and social services [50] making this a particularly salient issue.

An in-depth discussion of youth homelessness is beyond the scope of this paper; however, future resiliency studies among street-involved youth may want to focus on the role of housing in preventing IDU as our study found inadequate housing as a barrier to resiliency. Previous reports and research have suggested there are a variety of barriers at play for youth accessing adequate shelter: affordability, discrimination from landlords, alienation and isolation, lack of services for specific subgroups, lack of supports for 16–18 year olds, age of majority cut-off, lack of system flow and a lack of a provincial youth housing strategy [51]. Single-room occupancy hotels, many of which are located in the impoverished core of downtown Vancouver, have been reported as the only affordable and accessible housing option for at-risk youth; however, many youth resist this option as they view it as “giving up hope for a return to mainstream society” [52].

### Implications for practice

A number of findings from the YIP study may be useful for service providers and administrators to consider when undertaking service re-design and quality improvement activities: including street-involved adults and youth as peer outreach workers; providing opportunities for youth to be responsible to or for someone or something (jobs, pets, gardens, etc.); providing safe recreational spaces for street-youth, offering skill building and job placement programs, facilitating cultural (re) connection for Aboriginal street-youth, and finally, as learned by trial and error within the YIP research team, framing questions and leading conversations in a way that identifies strengths and opportunities, in addition to risks.

### Limitations

There are limitations to be noted when interpreting the findings presented in this paper. Previous research has shown that street youth are a heterogeneous population and that different sub-groups may have very different drug use patterns [49]. The youth participants in this study were referred by youth service provider organizations; therefore, youth who were currently not interacting with services were missed, and those “harder-to-reach” youth may hold different or unique perspectives regarding injection drug use initiation. Additionally, youth in the study were welcomed to share stories and experiences of their peers, as well as themselves, as a means of creating a safe space to participate; however, this may have introduced an element of misinterpretation or exaggeration in the data. The wide age range of the study participants (16–24) should also be considered when considering the perspectives shared; it is plausible that some of these youth, although at the time of the study were opposed to IDU, may progress to IDU later in life. This study did not consider the differences in perspectives between street-youth with different drug use patterns; ie: those using non-injection heroin, opioid prescription drugs and methamphetamines vs those using marijuana, alcohol and club drugs. Exploring any related differences in perspectives would be an interesting area of investigation for future research. Lastly, the YIP study and its findings are specific to the Metro Vancouver area, and care should be taken around the transferability of the findings to other geographic areas. As has previously been noted [53], there is a gap in research exploring the transition to IDU transition among rural populations; factors may differ from urban centers.

### Strengths

Engaging youth collaborators in the research process ensured relevance of the research. Youth collaborator engagement in the research included developing and revising the questioning guide, FG facilitation and note taking, a coding workshop and a knowledge translation event with service providers. These activities improved the reliability and validity of the results and enabled member checking by these experiential youth. The use of FG focus group observation notes also enabled cross checking and internal validation of the research findings.

## Conclusion

It is hoped that this paper will help service providers to recognize factors that deter IDU and help youth to navigate and negotiate for resources in their environments that promote healthy outcomes. People who work with at-risk youth should be mindful to not continually highlight risks and problems when conversing with street–involved youth but where possible, ask questions that encourage strength-identification and positive possibilities. Participants emphasized having personal goals and ties to social networks, supportive family and role models, and the need for safe and stable housing as key to resiliency and upon which prevention programs should be built. Gaining the perspectives of street-involved youth on factors that prevent IDU provides a complementary perspective to risk-based studies and encourages strength-based approaches for coaching and care of at-risk youth. Focusing on the personal, social and structural factors of resiliency at play in youths’ lives, and encouraging those protective factors, may interrupt the trajectory toward IDU.
